# The acute effects of cigarette smoke exposure on muscle fiber type dynamics in rats

**DOI:** 10.1371/journal.pone.0233523

**Published:** 2020-05-20

**Authors:** Kwok-Kuen Cheung, Timothy Kai-Hang Fung, Judith C. W. Mak, Sheung-Ying Cheung, Wanjia He, Joseph W. Leung, Benson W. M. Lau, Shirley P. C. Ngai

**Affiliations:** 1 Department of Rehabilitation Sciences, The Hong Kong Polytechnic University, Kowloon, Hong Kong; 2 Department of Medicine, The University of Hong Kong, Pok Fu Lum, Hong Kong; 3 Department of Pharmacology & Pharmacy, The University of Hong Kong, Pok Fu Lum, Hong Kong; University of Minnesota Medical School, UNITED STATES

## Abstract

Reduced exercise capacity is common in people with chronic obstructive pulmonary diseases (COPD) and chronic smokers and is suggested to be related to skeletal muscle dysfunction. Previous studies using human muscle biopsies have shown fiber-type shifting in chronic smokers particularly those with COPD. These results, however, are confounded with aging effects because people with COPD tend to be older. In the present study, we implemented an acute 7-day cigarette smoke-exposed model using Sprague-Dawley rats to evaluate early effects of cigarette smoking on soleus muscles. Rats (n = 5 per group) were randomly assigned to either a sham air (SA) or cigarette smoking (CS) groups of three different concentrations of total particulate matters (TPM) (CS_TPM2.5_, CS_TPM5_, CS_TPM10_). Significantly lower percentages of type I and higher type IIa fiber were detected in the soleus muscle in CS groups when compared with SA group. Of these, only CS_TMP10_ group exhibited significantly lower citrate synthase activity and higher muscle tumor necrosis factor-α level than that of SA group. Tumor necrosis factor-α level was correlated with the percentage of type I and IIa fibers. However, no significant between-group differences were found in fiber cross-sectional area, physical activities, or lung function assessments. In conclusion, acute smoking may directly trigger the onset of glycolytic fiber type shift in skeletal muscle independent of aging.

## Introduction

Cigarette Smoking has high global prevalence and a common risk factor of mortality for various diseases. [1] It is also the main contributor for developing chronic diseases that require long term medical care, such as chronic obstructive pulmonary disease (COPD). [2] In China alone, the prevalence of smoking in population aged 15 or above was 28% in 2010 [3] and the prevalence of COPD was 8% as reported in 2007. [4]

Reduced exercise capacity is often associated with both smoking and COPD. [5] For example, research has shown that asymptomatic smokers complain of frequent fatigue and reduced level of exercise capacity than non-smokers. [5] This finding suggests a potential link between exercise intolerance and smoking, even before the manifestation of any overt pulmonary conditions. [6] Thus, exercise intolerance in people with COPD may not be solely due to the intra-pulmonary changes associated with lung disease, but also by systemic extra-pulmonary changes induced by smoking. [7] Reduced exercise capacity has important health and quality of life implications, as it is correlated with both inactivity and premature mortality. [8] In view of the ample clinical evidence for exercise intolerance in COPD and non-COPD smokers, research attention has begun to focus on the pathophysiological mechanisms that may explain the effects of smoking on exercise capacity.

Peripheral skeletal muscle dysfunction has been hypothesized as one of the key factors contributing to reduced exercise capacity. [9–11] Such dysfunction is believed to be associated with the re-distribution of muscle fiber types and fiber atrophy in the locomotor muscles, most frequently seen in human quadriceps. [12] More specifically, muscle fibers have been observed to shift from type I oxidative muscle fibers to type II glycolytic muscle fibers. [12] In addition, muscle atrophy contributes to a reduction in the cross-sectional area of individual muscle fibers. [13] These extra-pulmonary changes could result in reduced muscle endurance and strength, ultimately leading to reduced exercise capacity.

A recent systematic review reported that smokers with COPD possessed significantly lower percentage (%) distribution of type I muscle fibers in their vastus lateralis muscle biopsy when compared with age-matched healthy controls. [12] It has been hypothesized that skeletal muscle dysfunction observed in people with COPD could be caused by multiple factors in addition to smoking. [9, 14] These factors include disease-induced pathological changes, inactivity, nutrition abnormality, corticosteroid therapy, and genetic factors. [9, 14] As mentioned previously, smoking is the primary contributing factor for the development of COPD. Individuals diagnosed with this condition usually report a long smoking history and have the disease diagnosed when they are older. This makes it difficult to determine if smoking induces early detrimental effects on locomotor muscle. Hence, the direct and early effects of smoking on muscle fiber type distribution and muscle atrophy, as measured by cross-sectional area, remain unclear.

Dysfunction in primary locomotor muscles has also been reported in chronic animal smoking models. [15, 16] For example, Nakatani and colleagues reported that rats exposed to an 8-week cigarette-smoke exposed model, when compared with control group, demonstrated a significantly lowered proportion of type I fiber and smaller fiber cross-sectional area in soleus, a primary muscle for locomotion in rodents. [15] Similar findings in soleus as well as other prime mover muscles e.g. gastrocnemius were observed in mice after a 32-week cigarette smoke-exposed model. [16] However, given the chronic nature of both smoking models, a coexistence of hypertension in rats and emphysema in mice were reported. [15, 16] It was speculated that such disease development may have played a role in the skeletal muscle dysfunctions observed. [15, 16] In short, while the extant findings are consistent with smoking having an immediate effect on fiber type distributions and muscle atrophy, whether or not smoking has a direct and early effect on muscle fiber type percentages and muscle atrophy has yet to be definitively confirmed.

The current study was designed to address this knowledge gap. Here we implemented an acute and short-duration animal smoking model to investigate if phenotypes such as fiber type shifting, muscle cross-sectional area reduction and reduced exercise activities observed in chronic smoking model also occur in this acute smoking model. As cigarette smoking is known to reduce the blood oxygen content that is believed to affect more on muscles containing high proportion of oxidative, slow muscle fibers, we therefore focused on soleus, which compose predominantly of type I fiber (80–90% in rodents). In addition, we also investigate whether alterations of skeletal muscle properties, if any, were associated with intra-pulmonary changes upon acute cigarette smoke exposure.

## Results

### Changes in respiratory variables and lung mechanics

**Fig 1:**
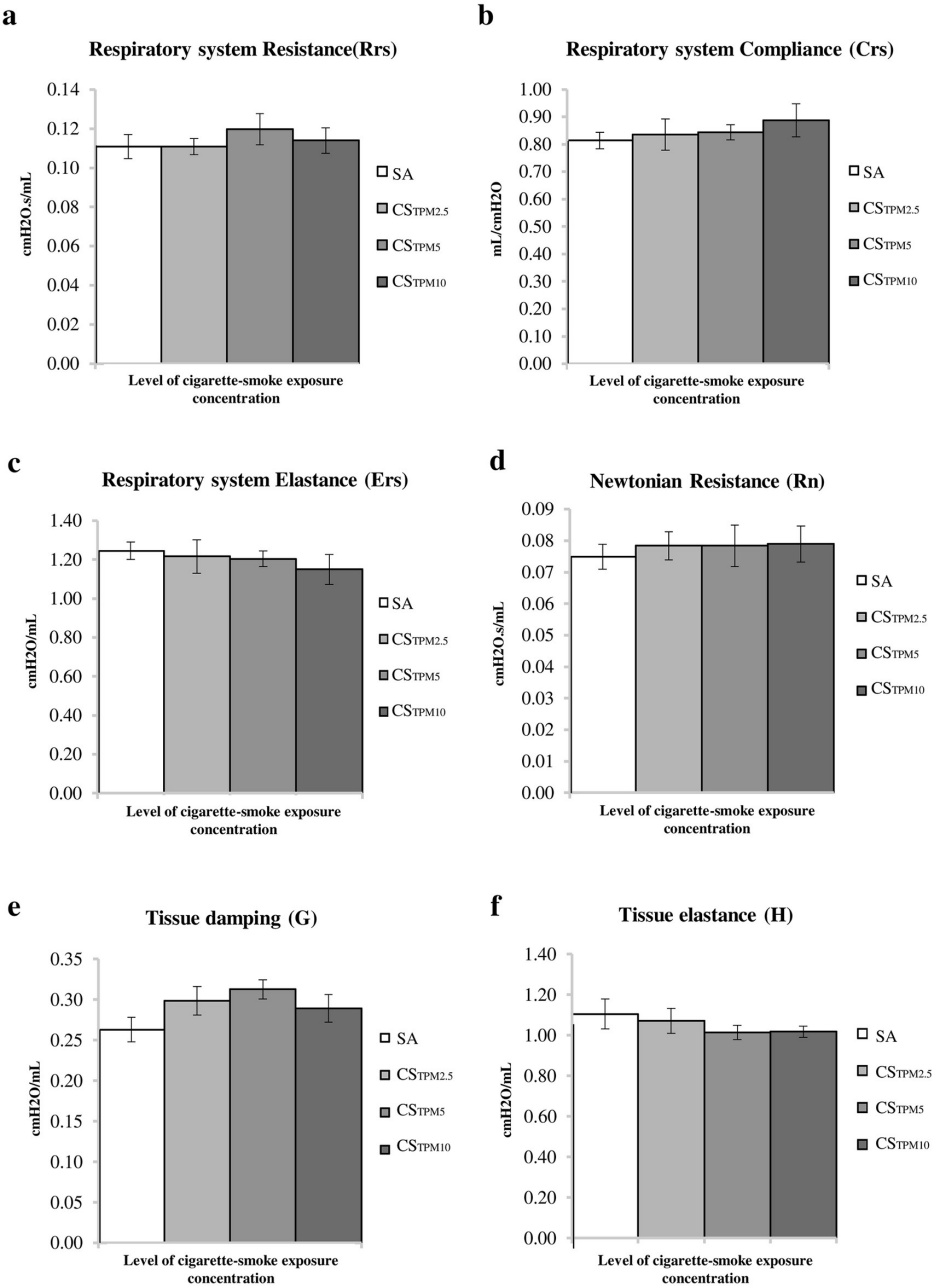
Various parameters of lung functions upon acute cigarette smoke exposure. Histogram summarizing the (a) respiratory system resistance, (b) respiratory system compliance, (c) respiratory system elastance, (d) newtonian resistance, (e) tissue damping and (f) tissue elastance (H) of the lung function parameter from SA group and CS group. All data are presented as mean ± SEM.

To address whether significant lung function impairment occurred following a 7-day acute smoking protocol, we investigated a battery of respiratory variables and lung mechanics. Our results showed that there was no significant change in main effects (time, group) or any Time x Group interactions for any of the respiratory variables among the four exposure groups of different TPM (Fig 1). Thus, the current acute smoking protocol did not induce any significant impairments in lung functions.

### Changes in muscle fiber types

**Fig 2:**
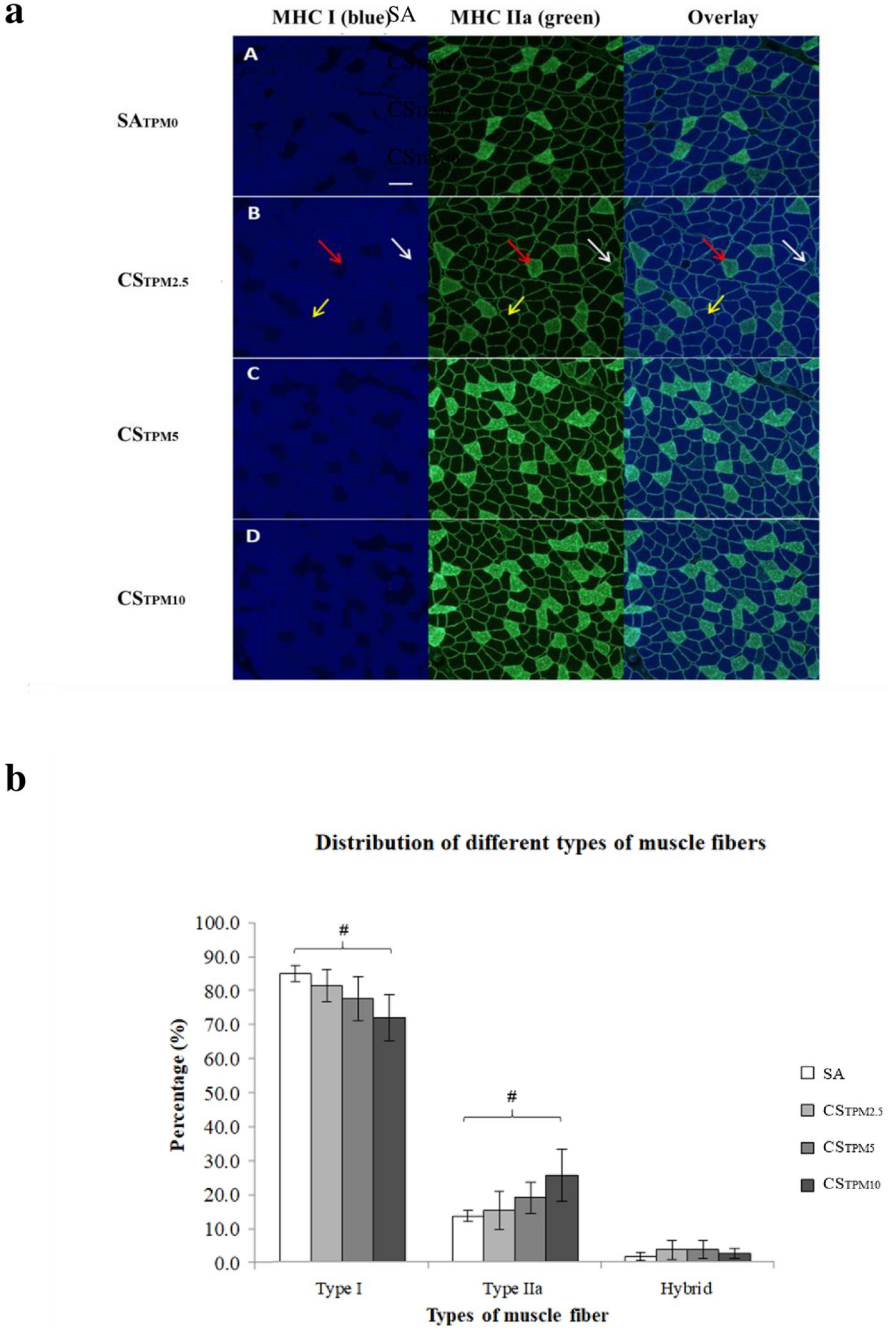
Muscle fiber types changes in soleus muscles upon acute cigarette smoke exposure. **(a**) Cross-sections of soleus muscles from SA (**A**), CS_TPM2.5_ (**B**), CS_TPM5_ (**C**) and CS_TPM10_ (**D**) groups showing immunoreactivity against MHC I (blue, left panel), MHCIIa (green, middle panel) and the overlaid images (right panel). Examples of type I fibers (yellow arrows), type IIa fibers (red arrows) and type I/IIa hybrid fibers (white arrows) were indicated. Scale bar = 100μm. (**b**) Histogram summarizing the percentage distribution of type I, type IIa and type I/IIa hybrid fibers in soleus from SA group and CS groups. All data are presented as mean ± SEM. * indicates *p*<0.05 for overall ANOVA comparison; ^#^ indicates *p*<0.05 in post-hoc analysis.

**Fig 3:**
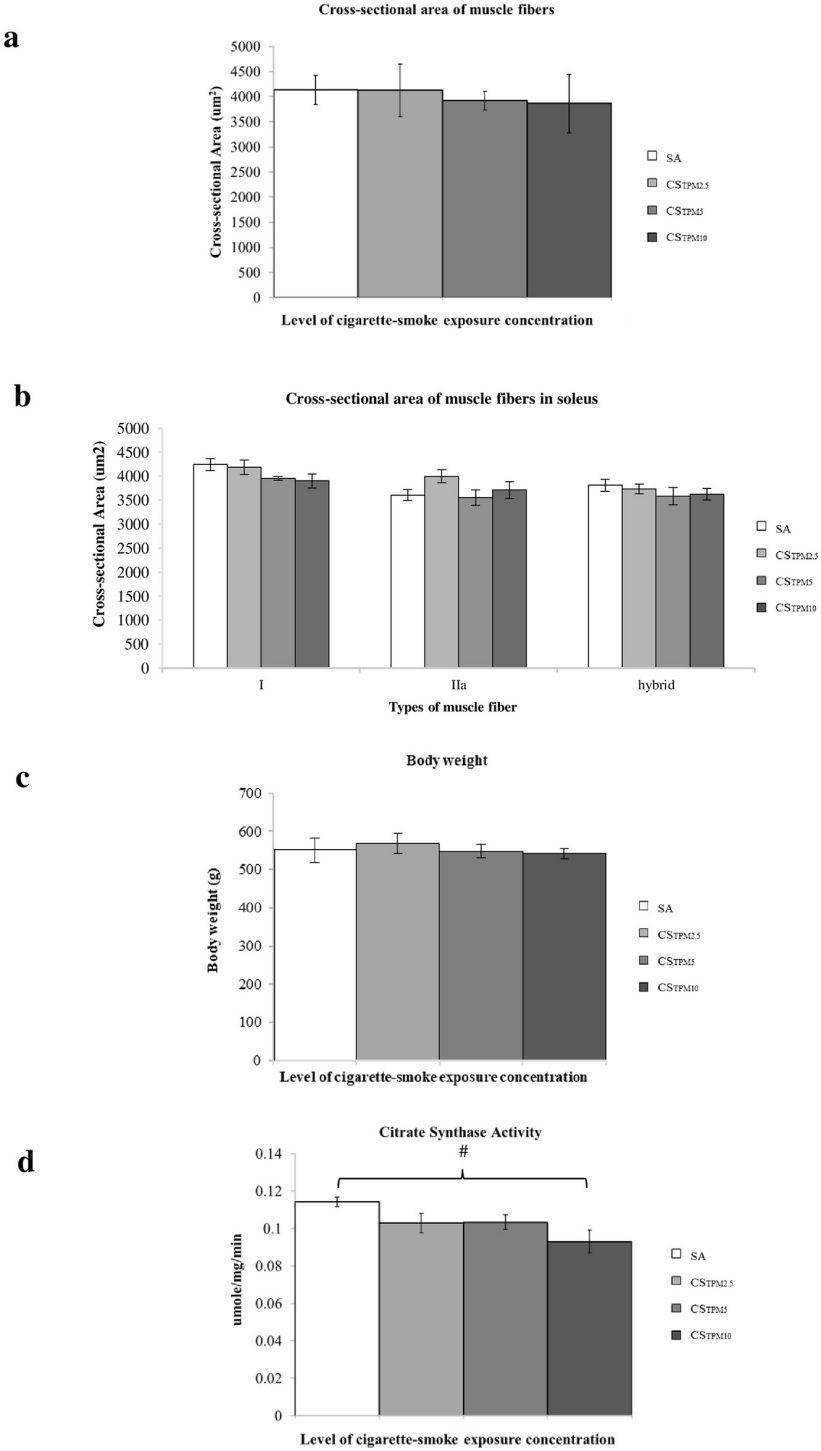
Muscle cross-sectional area, body weight and citrate synthase activity of soleus upon acute cigarette smoke exposure. Histogram summarizing the (**a**)) the overall cross-sectional area of soleus muscles, (**b**) the cross-sectional area of individual fiber types, (**c**) the body weight of rats and (**d**) the level of citrate synthase activity of soleus muscles from different SA group and CS groups. Data was presented as mean ± SEM. * indicates *p*<0.05 for overall ANOVA comparison; ^#^ indicates *p*<0.05 in post-hoc analysis.

To investigate if fiber type shifting occurred upon 7-day cigarette smoke exposure, multiple fluorescence immunohistochemistry was performed to map the distribution of different fiber types in soleus muscles and representative images were shown in Fig 2a. Soleus muscles mainly consisted of type 1 and type IIa fibers, constituting more than 96% of the total fibers (Fig 2b). There were very few any type IIb fibers present, yet a minor proportion of hybrid fibers co-expressing type I and IIa MHC was detected. Interestingly, the percentage of type I fibers in the soleus decreased with smoking (in all CS groups) when compared to SA control groups. In addition, the percentages of type IIa fibers in all CS groups were higher than that of the SA group. The percentage of type I fibers followed a dose-dependent reduction (r = -0.71, *p* = 0.001) with increasing concentration of TPM, with a concomitant increase in type IIa fibers with increasing TPM (r = 0.69, *p* = 0.001) (Fig 2b). Significant between-group differences were reported among SA, CS_TPM2.5_, CS_TPM5_ and CS_TPM10_ (*p*<0.05). Post-hoc analysis revealed that among groups with different concentration of TPM, only the CS_TPM10_ group (type I: 71.9%; type IIa: 25.5%), but not the groups of lower doses, exhibited a significant difference in the percentage of type I and type IIa fibers with control SA group (type I: 83.7%; type IIa: 14.1%). Despite a significant difference was found in fiber type distribution, there was no difference in terms of fiber cross-sectional area measured (Fig 3a and 3b). Likewise, no significant changes in body weight was observed after CS exposure (Fig 3c).

We next compared the citrate synthase activity between CS and SA groups. The citrate synthase is a common enzyme for energy generation through citric acid cycle and a routine biomarker of aerobic capacity in skeletal muscle. The activity of the citrate synthase is a metabolic marker that indicates the mitochondrial content and the oxidative capacity of muscles. One way ANOVA revealed significant between-group differences in citrate synthase activities (*p* = 0.042) while post-hoc analysis showed that the CS_TPM10_ groups was significantly lower than that of the SA group (*p* = 0.033). Furthermore, the average citrate synthase activities of CS_TPM2.5_ and CS_TPM5_ were both lower than the SA _TPM0_ group (Fig 3d).

### Changes in physical function

**Fig 4:**
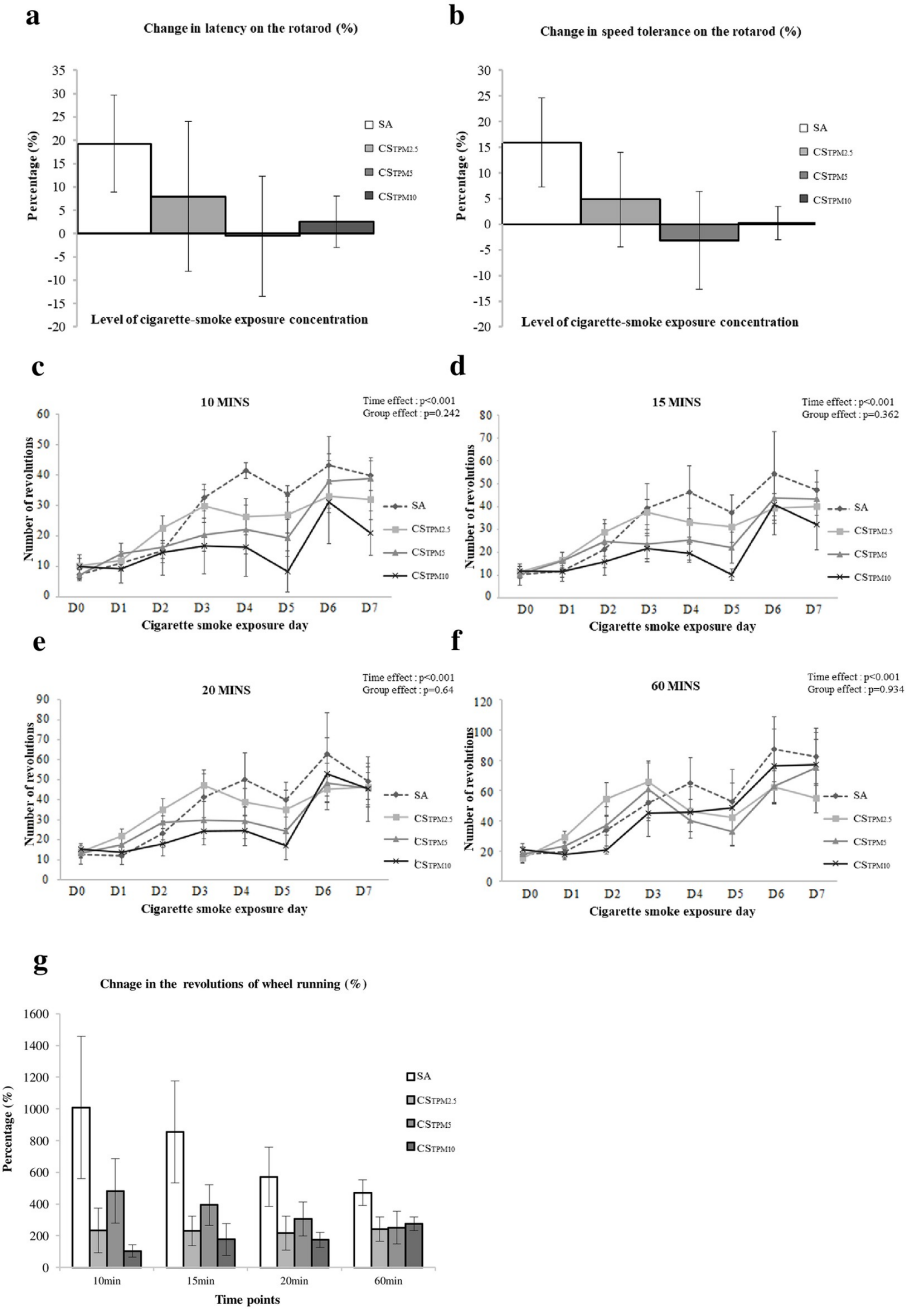
Changes in physical activities after acute cigarette smoking exposure. Data summarizing the percentage in (**a**) latency and (**b**) speed tolerance on the rotarod, and the graphs summarizing daily voluntary activities in terms of the revolutions in (**c**) the first 10 minutes, (**d**) the first 15 minutes, (**e**) the first 20 minutes, and (**f**) the entire 60 minutes recorded using voluntary wheel, between SA and other smoking exposure groups. (**g**) Histogram summarizing the percentage changes in revolutions of wheel running recorded at various time points. Data was presented as mean ± SEM. D indicates the day of experiment.

We have compared the performances on rotarod test and voluntary wheel running between SA group and different CS groups after 7-day cigarette smoke exposure. Results from the rotarod performance assessment showed no statistical difference in the latency or the speed tolerance between SA group and different CS groups (*p*>0.05), although as can be seen in Fig 4a & 4b, the SA group mice tends to perform better than any of the CS groups. Besides, we observed a tendency of an increase in running activity in the SA mice, relative to the CS mice from day 4 onwards till day 7, despite statistically insignificant (Fig 4c–4f). We then compared the percentage change of the revolutions run between day 0 and day 7. Regardless of the intervals analysed (whether it was the first 10 minutes, 15 minutes, 20 minutes or even the entire 60 minutes of running), SA group always demonstrated greater progress over the 7-day treatment periods. We also compared the percentage changes between day 0 and day 7 in terms of the total revolutions run per hour between SA group and CS groups. SA group showed a better improvement in the total revolutions run as compared to all CS groups, although the difference did not reach statistical significance (Fig 4g).

### Changes in inflammatory cytokines

**Fig 5:**
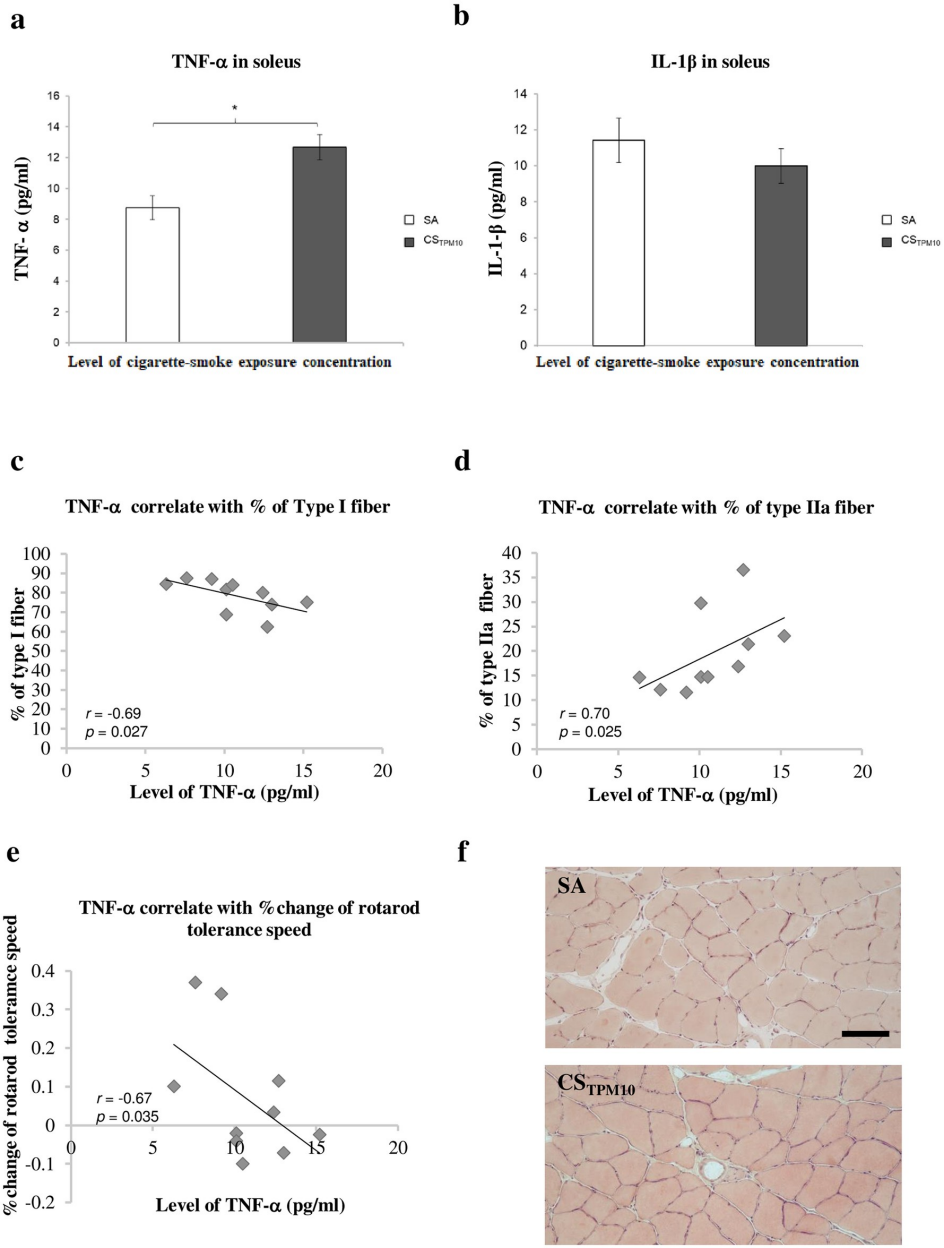
TNF-α and IL-1β expression in soleus muscles after acute cigarette smoking exposure. Bar chart summarizing the level of (**a**) TNF-α and (**b**) IL-1β in soleus muscle of SA and CS_TPM10_ groups. Data was presented as mean ± SEM. * indicates between group difference with *p*<0.05 (t-test). (**c-e**) Graphs illustrating the correlation of level of TNF-α expression with (**c**) percentage of type I fiber, (**d**) percentage of type IIa fiber and (**e**) percentage change of rotarod tolerance speed. (f) Representative H & E stained images showing the morphology of soleus muscles for SA group (upper panel) and CS_TPM10_ (lower panel) groups. Values of *r* and *p* were shown in respective graphs. Scale bar = 100μm.

As an indication of local inflammation in soleus muscles, we looked at the levels of inflammatory cytokines TNF-α and IL-1β were increased after acute cigarette smoke exposure. Results from immunoassay indicated that statistically significant upregulation in TNF-α was observed in the soleus muscle of CS_10TPM_ as compared to control SA (Fig 5a). However, quantitative alterations in IL-1β level in soleus were not observed between SA group and CS_10TPM_ group (*p* = 0.008) (Fig 5b). Furthermore, comparison between CS_10TPM_ and SA group have shown that increase in TNF-α level observed in CS_10TPM_ correlated with reduction in type I fibers (r = -0.69; *p* = 0.027) and increment in type IIa fibers (r = 0.70; p = 0.025), as well as with a decrease in rotarod speed tolerance (r = -0.67; *p* = 0.035) (Fig 5c–5e). Despite significant difference in TNF-α expression, H & E staining revealed absence of obvious inflammatory cell infiltration neither in the SA nor CS_TPM10_ groups (Fig 5f).

## Discussion

Cigarette smoking is known to cause direct damage on lung tissue and leads to development of numerous lung diseases. [17–20] It is generally believed that all the adverse effects of cigarette smoking on different organ systems, whether pulmonary-related or not, is a consequence of chronic respiratory dysfunction. The findings from the present study offer another perspective, in which cigarette smoking may also induce acute effects on muscles distinct from its effects on respiratory functions. In our experimental model, as the TPM concentration increased, the level of serum cotinine after 7-day acute cigarette smoking increased. The results confirmed that the rats were indeed exposed to cigarette smoking due to accumulation of cotinine in the serum (S1 Fig). Despite a high level of CS exposure, acute cigarette exposure did not induce significant changes in the respiratory variables, suggesting that any effects of smoking observed on other systems are likely due to mechanisms that are independent of pulmonary function decline.

The fiber type distribution shift of soleus observed in the current 7-day acute cigarette-smoke exposed model is consistent with the findings reported in other chronic cigarette smoke exposed human and animal models. For example, one previous human biopsy study found significantly lower percentage distribution of type I muscle fibers and higher percentage distribution of type IIa muscle fibers in the vastus lateralis muscle of smokers with COPD in comparison with age-matched healthy controls. [13] In animal studies using chronic cigarette-smoke exposed models, similar findings were reported in soleus muscles of rats and mice after an 8-week and 32-week cigarette smoke exposure, respectively. [15, 16] These findings support the potential glycolytic shift in fiber type of locomotor muscle of human and animals after prolonged periods of smoking. The same shift demonstrated in this study suggests the possibility that the impact of cigarette smoking on muscle fiber redistribution occurs even at the early stage of smoking independent of overt respiratory pathologies.

It is known that type I slow-twitch fibers undergo oxidative phosphorylation to obtain energy during muscle contraction, [21] and they require. comparatively more oxygen consumption than type II fast-twitch fibers that rely on glycolytic pathway for energy production. Carbon monoxide (CO), one of the main constituents of inhaled cigarette smoke that demonstrate 200-fold greater affinity than O_2_ for hemoglobin, can reduce the oxygen (O_2_) carrying capacity of blood as well as diminish O_2_ delivery to the mitochondria of active muscles and impairs cellular metabolism. [6] The level of COHb had increased up to 9% COHb for chronic smokers, which was comparable to a hypoxemic condition. [22] Mitochondrial respiratory chain functions have been shown to be impaired by tar and cyanide along with the different free radicals that are present in cigarette smoke. [23, 24] Taken together, these findings indicate that the inhalation of cigarette smoke leads to reduced availability of O_2_ for energy production and damage to mitochondria of muscle fibers.

In response to the lack of availability of O_2_, the muscle fibers may therefore undergo anaerobic adaptation and morphological modification, resulting in the glycolytic shift of type I oxidative muscle fibers [6], which consists of greater proportion of type 1 oxidative fibers responsible for endurance activities. [25] This anaerobic adaptation increases the proportion of the fast-twitch type II fiber that may alters the muscle activity in oxidative glycolysis cycle. Citrate synthase activity is a common biomarker of oxidative capacity and mitochondrial density in muscle. [26] Our results showed that citrate synthase activity was significantly reduced in acute 7-day cigarette smoke exposure at the high TPM concentration group, supporting the notion that cigarette smoke exposure may shift the muscle fibers from aerobic capacity to anaerobic respiration system for muscle contraction. This also could explain the decreased muscle endurance leading to reduced exercise capacity presented in smokers. [9] Our data on voluntary wheel running tend to support this notion. Regardless of the time spent on running or the total revolutions run, our control animals tended to demonstrate a better performance, particularly in the first 10–15 minutes, compared to that of the cigarette smoking groups, despite the difference not reaching statistically significant. Considering that the smoking exposure period was only 7 days, it seems like that more significant impacts on physical activities, associated with a higher proportion of type I fiber reduction, is expected with longer duration of cigarette smoking.

In contrast to previously reported human and animal chronic smoking models [27, 28], this acute smoking model yielded no significant reduction in the fiber cross-sectional area of soleus Smoking-induced muscle fiber atrophy is hypothesized to be the net result of a complex process that involves muscle proteolysis activation through the nuclear factor-κB (NF-κB) pathway [29], and recent studies suggested that the NF-κB pathway may possibly be triggered by TNF-ɑ, a pro-inflammatory cytokine which has been implicated in disuse muscle atrophy and is generally found in smokers. [30, 31] This is also consistent with our current finding that upregulation of TNF-ɑ was detected in this acute cigarette exposure model. TNF- α, apart from being a circulating proinflammatory cytokine produced by both neutrophils and macrophages, can also be released by skeletal myocytes that serve as an endogenous mediator for muscle adaptation upon injury and the subsequent muscle regeneration. [32–35] On the other hand, we did not observe an increase in another major proinflammatory cytokine IL-1β, suggesting that either the thresholds for activation of IL-1β and TNF-α were different upon acute smoking, or an increase TNF-α level in muscles might not necessarily be related to inflammation but instead could be an adaptive response of muscle towards smoking exposure. Yet, our data did not rule out the possibility that cigarette smoking triggers muscle inflammation. In fact, Chan et al [36] demonstrated that cigarette smoking exacerbated muscle inflammation upon subsequent muscle injury, despite they did not observe upregulation of inflammatory cytokines even after 8-week of cigarette smoking, probably because they looked at mRNA transcripts instead of protein levels. Nevertheless, significant correlations between TNF-α and fiber type shifting suggesting the involvement of TNF-α pathway in the process of fiber derangement. Krüger and coworkers demonstrated that a gradual decrease in soleus fiber cross-sectional area was time-dependent and that the difference did not reach significance until 32 weeks of chronic cigarette-smoke exposure in mice. [16] Therefore, it is possible that smoke exposure time in this acute smoking model is not sufficient to fully induce the underlying physiological and morphological changes in muscle fibers.

This study focused on the morphological investigation of muscle fibers while evaluating the feasibility of an acute rat smoking model. To have a comprehensive understanding on the underlying mechanisms and functional impact of smoking-associated muscle fiber changes, investigation on the mechanistic and behavioral aspects may be necessary. For example, measurements of the entire exercise performance using home-cage approach would allow a better interpretation of the effect of smoking on physical activities. Besides, effects of cessation of cigarette smoking after the acute exposure should also be investigated to demonstrate whether the process of fiber type shifting is entirely reversible.

## Summary and conclusion

By implementing an acute, 7-day rat smoking model, this study demonstrated that smoking may have a direct and early role in the glycolytic fiber type shift in locomotor muscle. Such shift is well documented to decrease muscle endurance which may contribute to reduced exercise capacity. [37] It is worth noting also that reduced physical activities further accelerate lung function decline. [28] Knowing the reversible nature of this shift and the potential vicious cycle, early endurance training is recommended clinically to preserve exercise capacity in smokers who may be affected by health issues that limit their activity level. However, to fully understand the underlying physiological mechanisms between smoking and exercise tolerance, more comprehensive studies investigating the mechanistic and behavioral aspects of muscular dysfunction are warranted. Nevertheless, the present study provided a feasible, short-duration, acute smoking model that can serve as a starting platform for future full-scale studies to build upon current findings.

## Experimental procedures

### Animals and acute smoking model

Sixteen-week old male Sprague-Dawley (SD) rats from the same litter were obtained from centralized animal facilities of the Hong Kong Polytechnic University. All animal care and experimental procedures were approved by the Animal Subject Ethics SubCommittee of the Hong Kong Polytechnic University (ASESC Case #15-16/15-RS-R-OTHERS), and were performed in accordance with the relevant guidelines and regulations. The rats were housed in groups of two to three at a temperature of 21°C and 60% relative humidity under a 12-hour light-dark cycle. They were supplied with a standard laboratory diet and sterile water *ad libitum*. The rats were randomly allocated into 4 groups (n = 5 per group) which were exposed to cigarette smoking as described previously with some modifications. [38, 39] The smoking apparatus is composed of two peristaltic pumps (A & P Instrument), 0.5L mix chamber and 32L exposure chamber. The fresh air (960ml/min) and cigarette smoke (Camel; filter-removed, R.J. Reynolds, Winston-Salem, NC, USA) (40ml/min) were simultaneously pumped into the mix chamber, after which the gas passed through a regulator for controlling the amount of mixed gas before going into the exposure chamber through a CEL-712 Microdust Pro Real-time Dust Monitor (Casella, UK), where the concentration of TPM was recorded. Different concentrations of total particulate matter (TPM): SA (sham air, equivalent to TPM0); CS_TPM2.5_ (CS at 2.5g/m^3^, approximately equivalent to 4% CS as reported previously) [38, 39]; CS_TPM5_ (CS at 5g/m^3^); CS_TPM10_ (CS at 10g/m^3^) cigarette smoke (CS) were adjusted. The rats were exposed to cigarette smoke at a particular TPM inside the chamber for 1 hr, twice daily with 1hr interval between exposures for 7 consecutive days.

### Outcome measures

#### Physical activities

Motor coordination was assessed by measuring the time the rats stayed on a rotarod (Panlab, Harvard apparatus, USA) before and after 7-day smoking exposure. In addition, to measure voluntary activity level, the rats were also allowed to run daily on a voluntary wheel for 7 days. The number of revolutions/hour that the rats ran on the voluntary wheel was recorded.

#### Evaluation of lung mechanics

Twenty-four hours after the end of 7-day protocol, rats were anesthetized with an intraperitoneal injection of pentobarbitone (50mg/kg body weight). They were then tracheostomized and ventilated with an average breathing frequency of 90 breaths/min using Flexivent (SCIREQ, Montreal, Canada). The lungs were inflated to allow the alveolar holding of 30cm H_2_O pressure to achieve total lung capacity state. After that, the respiratory system resistance (Rrs), elastance (Ers), and compliance (Crs) were measured in a single frequency forced oscillation manoeuvre. The newtonian resistance (Rn), tissue damping (G) and tissue elastance (H), were also obtained in a broadband forced oscillation manoeuvre. Each measurement cycle performed three times. The coefficient of determination of each of the measured parameter was higher than 0.95. Average values for each lung mechanic measure were computed and was used in the analyses.

#### Animal sacrifice and tissue processing

After the evaluation of lung mechanics, animals were sacrificed with an overdose of intraperitoneal injection of pentobarbitone (100mg/kg body weight). Immediately after sacrifice, the soleus muscles of the right hindlimb were carefully isolated and snap-frozen in liquid nitrogen and stored at -80°C for enzyme assays and ELISA assessments, while the soleus muscles of the contralateral hindlimb were embedded in O.C.T.^™^ medium (Leica Biosystems, Germany) under liquid nitrogen. Cross-sections of 7μm thickness were cut from the muscle mid-belly and were stored at -80°C until the subsequent histological analyses.

#### Muscle cross-sectional area measurement

The 7μm-thick cross-sections were air-dried, fixed in 4% paraformaldehyde in Phosphate Buffered Saline (PBS, pH 7.4), rinsed, and stained with hematoxylin and eosin. The stained sections were dehydrated with graded alcohol and cleared in xylene before mounting. Sections were examined with a light microscope (20X objective magnification) and images were acquired using a Spot digital camera. Measurements of the muscle fiber cross-sectional area (in μm^2^) were obtained using Image J imaging software (National Institutes of Health, USA).

#### Multiple fluorescence immunohistochemical analysis of muscle fiber types

The sections were air-dried, fixed in ice-cold 4% paraformaldehyde (in PBS) and washed in PBS. Non-specific binding was blocked with 10% normal goat serum in PBS for 30 minutes. For immunofluorescence analysis of myosin heavy chain (MHC), sections were first incubated with specific primary monoclonal antibodies (BA-F8 for MHCI, SC-71 for MHCIIa and BF-F3 for MHCIIb, all from Developmental Studies Hybridoma Bank, University of Iowa, USA) for 2 hours at room temperature, washed, followed by incubation with iso-type specific Alexa Fluor-conjugated secondary antibodies (Molecular Probes, Thermo Fisher Scientific, USA) for 1 hour at room temperature in dark. Sections were mounted with antifade mounting medium (DAKO) and visualized under fluorescence microscope (40X objective, Eclipse 80i; Nikon Instrument Inc, Japan). Images were captured using Spot Advanced software (Diagnostic Instruments Inc, USA). Individual images were taken across the entire cross-section and assembled into a composite panoramic image with Microsoft Image Composite Editor (Microsoft). All fibers within the entire muscle/cross-section were characterized for fiber type analyses. We then computed the percentages of each type of muscle fiber.

#### Muscle preparation and citrate synthase (CS) activity assay

The snap frozen soleus muscle samples were homogenized in ice-cold assay buffer and kept in ice for 10 minutes before centrifugation at 10,000g at 4°C for 10 minutes. The supernatant was collected for the citrate synthase (CS) assay using the Citrate Synthase Assay Kit (Sigma, St. Louis, USA). The enzyme assay was performed according to the manufacturer’s protocol.

#### Inflammatory cytokine assessment

Dissected soleus muscles were snap frozen in liquid nitrogen and stored in -80C freezer until used. Upon thawing, tissues were homogenized with RIPA buffer (abcam, Cambridge, UK) containing protease inhibitor cocktail (Roche applied sciences, Germany). The total protein content of each sample was measured using Bradford Assay (BioRad, Hercules, USA). Equal amount of protein (100μg) for each sample was used for measuring the levels of TNF-α & IL-1β by specific ELISA kits (R&D Systems, USA) according to manufacturer instructions.

### Data analyses

All data were presented as mean ± standard error of the mean (SEM) unless otherwise indicated. One-way ANOVA was used to examine between-group differences, followed by post-hoc Bonferroni-corrected univariate analyses if found to be significant. Changes in physical function (i.e. time stayed on rotarod and number of revolution of voluntary wheel activity) and respiratory variables were compared by using two-way repeated measure ANOVA (within group factor: time; between group factor: group). Pearson’s correlation coefficients were computed to estimate the associations between amount of CS exposure and observed outcomes. Statistical significance was set at p<0.05. Data were analyzed by IBM^®^ SPSS^®^ statistics (SPSS, Version 23.0, Chicago, IL, USA).

## Supporting information

S1 Fig (PDF)
