# Supplementary material for: The acute effects of cigarette smoke exposure on muscle fiber type dynamics in rats
Source: PLoS One. 2020 May 20;15(5):e0233523. doi: 10.1371/journal.pone.0233523 (PMC7239437; doi:10.1371/journal.pone.0233523)
Supplement: S1 Fig — (PDF) [file pone.0233523.s001.pdf]

The Acute Effects of Cigarette Smoke Exposure on Muscle Fiber Type Dynamics in Rats

#Kwok-Kuen CHEUNG,[1] #Timothy K FUNG,[1] Judith CW MAK,[2,3] Sheung-Ying CHEUNG,[1] Wanjia HE,[1] Joseph W LEUNG,[1] Benson WM LAU,[1*] Shirley PC NGAI[1*]

Supplementary Method

Experimental Procedure of serum cotinine measurement

Blood samples were collected from cardiac when rats sacrificed. Then blood was processed by centrifugation at 1900 × g for 15 min and serum was collected as supernatant and stored at −80 °C . The concentration of cotinine was determined by commercially available ELISA kit (Cotinine Direct ELISA, BioQuant, San Diego, CA) according to the manufacturer's instructions. The lower limit of detection was 1 ng/mL.

Supplementary Figure 1

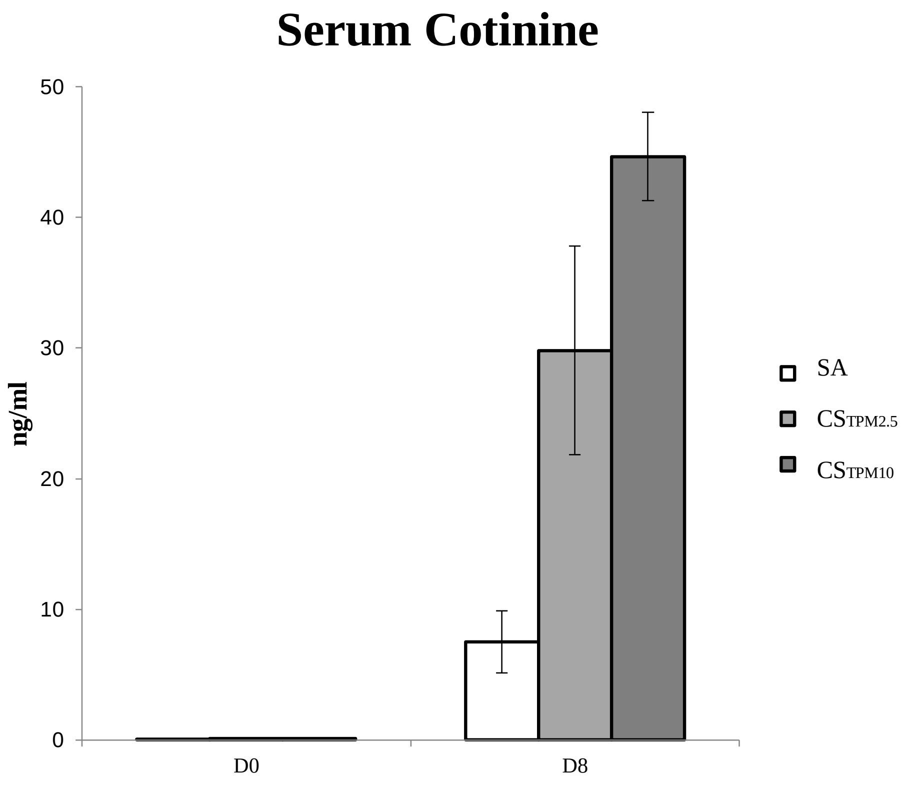

Histogram summarizing the level of serum cotinine of SA and CS groups at day 0 (D0) and day 8 (D8). Data was presented as mean ± SEM.
